# A Pragmatic Guide to Enrichment Strategies for Mass Spectrometry–Based Glycoproteomics

**DOI:** 10.1074/mcp.R120.002277

**Published:** 2020-12-20

**Authors:** Nicholas M. Riley, Carolyn R. Bertozzi, Sharon J. Pitteri

**Affiliations:** 1 Department of Chemistry, Stanford University, Stanford, California, USA; 2 Howard Hughes Medical Institute, Stanford, California, USA; 3 Department of Radiology, Canary Center at Stanford for Cancer Early Detection, Stanford University School of Medicine, Palo Alto, California, USA

**Keywords:** Glycosylation, glycopeptides, glycoproteomics, mass spectrometry, enrichment, chemical biology, affinity chromatography, lectins, hydrophilic interaction chromatography (HILIC), strong anion exchange electrostatic repulsion hydrophilic interaction chromatography (SAX-ERLIC), AAL, *Aleuria aurantia* lectin, AX, anion exchange, ConA, concanavalin A, CuAAC, *i.e.*, “click” chemistry, copper-catalyzed azide-alkyne cycloadditions, DIA, data-independent acquisition, ECD, electron capture dissociation, ERLIC, electrostatic repulsion-hydrophilic interaction chromatography, ETD, electron transfer dissociation, GalT1, β-1,4-galactosyltransferase 1, HCD, higher-energy collisional dissociation, HILIC, hydrophilic interaction chromatography, IMAC, immobilized metal affinity chromatography, IMS, ion mobility spectrometry, LAC, lectin affinity chromatography, M-LAC, multi-lectin affinity chromatography, M6P, mannose-6-phosphate, ManNAz, *N*-azidoacetylmannosamine, MAX, mixed-mode strong anion exchange, MOAC, metal oxide affinity chromatography, MS, mass spectrometry, Neu5Ac, *N*-acetylneuraminic acid, Neu5Gc, *N*-glycolylneuraminic acid, PGC, porous graphitic carbon, PTMs, post-translational modifications, RCA, ricinus communis agglutinin, SAX, strong anion exchange, Siglecs, sialic acid–binding immunoglobulin-type lectins, SPAAC, *i.e.*, “copper-free click” chemistry, strain-promoted azide-alkyne cycloaddition, SPE, solid-phase extraction, WAX, weak anion exchange, WGA, wheat germ agglutinin, ZIC-HILIC, zwitterionic HILIC

## Abstract

Glycosylation is a prevalent, yet heterogeneous modification with a broad range of implications in molecular biology. This heterogeneity precludes enrichment strategies that can be universally beneficial for all glycan classes. Thus, choice of enrichment strategy has profound implications on experimental outcomes. Here we review common enrichment strategies used in modern mass spectrometry–based glycoproteomic experiments, including lectins and other affinity chromatographies, hydrophilic interaction chromatography and its derivatives, porous graphitic carbon, reversible and irreversible chemical coupling strategies, and chemical biology tools that often leverage bioorthogonal handles. Interest in glycoproteomics continues to surge as mass spectrometry instrumentation and software improve, so this review aims to help equip researchers with the necessary information to choose appropriate enrichment strategies that best complement these efforts.

## Highlights

• Glycosylation is complex and often requires enrichment prior to analysis
• Review of common enrichment strategies for mass spectrometry–based glycoproteomics
• Enrichment methods have practical considerations and experimental implications
• Appropriate enrichment strategies will complement developments in mass spectrometry

## Graphical Abstract

**Figure:**
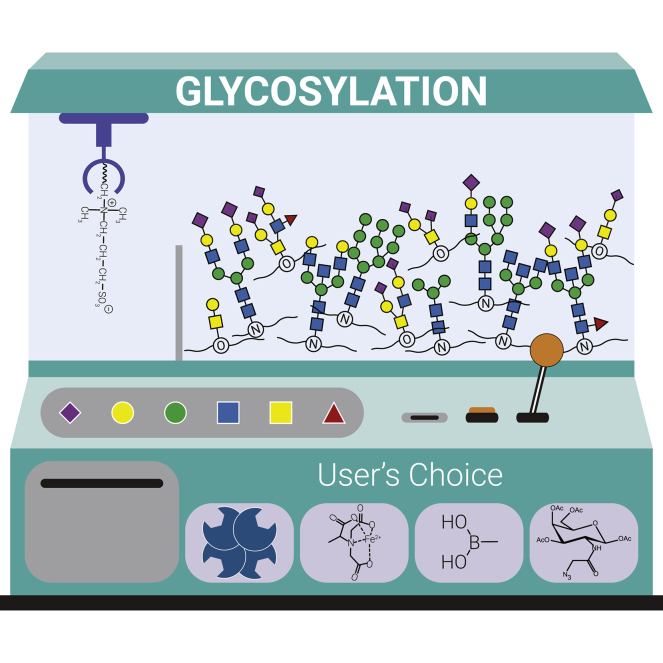

## In Brief

Interest in mass spectrometry–based glycoproteomics analysis is increasing because of recent advances in instrumentation and data analysis tools. Such studies can provide a wealth of information across a wide spectrum of glycan classes and biological systems. However, many studies require the choice of an enrichment strategy for glycosylated species prior to analysis to obtain the maximum amount of analytical information. Here, common enrichment strategies are reviewed with strengths and weaknesses, and the practical considerations for various methods are discussed.

**Fig. 1:**
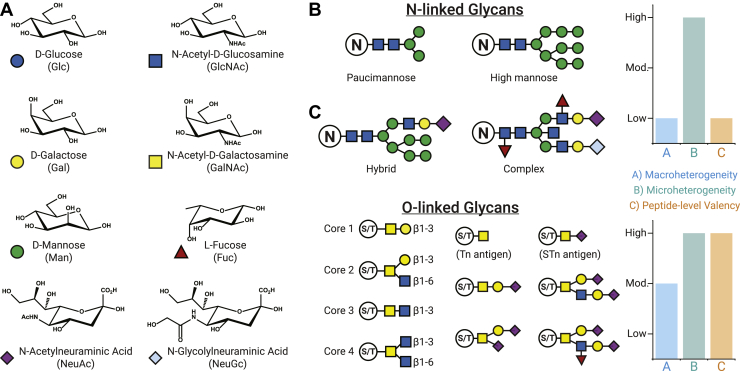
**Common units in mammalian glycosylation.***A*, common monosaccharides found in mammalian N- and O-glycans. *B*, N-glycans can be broadly categorized into four classes: paucimannose, high mannose, hybrid, and complex types. *C*, O-glycans can show a high degree of heterogeneity. Here, core 1 to 4 structures of mucin-type O-GalNAc glycans are shown, which can be further elaborated. Common examples of mucin-type O-glycans are provided. Graphs at the right comment generally on the degree of macroheterogeneity, microheterogeneity, and peptide-level valency (*i.e.*, how many glycosites commonly occur in a given glycopeptide) to be expected for both N- and O-glycosites.

Mass spectrometry (MS)-based methods are the premier tool for characterizing protein glycosylation, a universal co- and posttranslational modification that exists in all known domains of life (1). This super class of modifications is heterogeneous with several levels of classifications, each of which requires specific analytical considerations (2, 3, 4, 5, 6). Glycosylation is primarily defined by the nature of the covalent linkage of mono- or oligosaccharides (*i.e.*, glycans) to polypeptide backbones, usually through nitrogen or oxygen atoms on amino acid side chains (N- and O-glycans, respectively). Hundreds of monosaccharides exist, but only a subset of these are found in commonly observed glycans (7), as shown in Figure 1. Unlike the protein substrates they modify, glycans are not encoded directly in the genome. Instead, their structures are governed by numerous competing and sequentially acting glycosyltransferases and glycosidases that give rise to a diverse pool of glycans constructed from a large number of combinatorial possibilities (8, 9). Glycan expression is also dynamically regulated in response to environmental cues, making precise prediction of glycan structures difficult even with full knowledge of relevant gene products (10, 11, 12).

N-glycans and O-glycans also differ significantly in their core structures and the processing steps that define them (13). N-glycans are most often linked through *N*-acetylglucosamine (GlcNAc) at a consensus peptide motif: N-X-S/T, where X is not proline (14, 15, 16). Mammalian N-glycans also share a common pentasaccharide core that can then be differentiated into high-mannose, complex, and hybrid types that vary in the connectivity (branching) and composition of their monosaccharide substituents (Fig. 1*B*). Paucimannose N-glycan structures that are subsets of the pentasaccharide core also exist (17). O-Glycosylation, on the other hand, is defined by a number of different monosaccharide linkages, mainly to serine and threonine residues (18, 19, 20). Two major classes are intracellular O-GlcNAc, defined by β-GlcNAc with little elongation, and extracellular mucin-type O-GalNAc, defined by an initiating α-GalNAc that can be further decorated into several different core structures (Fig. 1*C*). O-Fucose, O-mannose, O-glucose, and O-xylose classes of O-glycosylation also exist, although most studies currently focus on the O-GlcNAc and O-GalNAc varieties. C- and S-glycosylation through carbon and sulfur atoms on tryptophan and cysteine residues are also known (21, 22, 23, 24, 25), but most of the focus here will remain on N- and O-glycosylation in higher mammals. Discussion of glycosylation in other systems, *e.g.*, prokaryotes, plants, and, insects can be found elsewhere (26, 27, 28, 29, 30).

Beyond these differences in classes, macroheterogeneity and microheterogeneity are integral facets of glycosylation. Macroheterogeneity refers to occupancy, or the presence or absence of glycans at a glycosite, and this level of regulation is common among all posttranslational modifications (PTMs). Microheterogeneity is a more fascinating, yet more confounding phenomenon of glycosylation where individual glycosites can harbor a range of glycan structural varieties, giving rise to numerous glycoforms that can exist for a single glycosite, much less a single gene product. Differences in glycan composition contribute greatly to glycosite heterogeneity, but structural isomers also contribute, such as conformational isomers (α *versus* β) or linkage differences (*e.g.*, α2,3-linked *versus* α2,6-linked sialic acid). Microheterogeneity can vary glycosite by glycosite or can be somewhat uniform across a given glycoprotein, and differences in glycan structures at a single glycosite can alter biological relevance (31, 32, 33, 34, 35, 36). The term “metaheterogeneity” has recently been proposed to describe these levels of variation across glycosites of a given protein (37). Thus, several degrees of characterization exist in glycoproteomic studies, which can range from detection of glycosites following removal of their modifying glycans, to characterization of intact glycopeptides and glycoproteins to interrogate different glycoforms of modified sequences.

Heterogeneity differs for N- and O-glycan classes (Fig. 1, *B*–*C*). The diversity of N-glycan structures can lead to high microheterogeneity at a given site, but the presence of a glycan at a given glycosite is less dynamic even if the glycan itself differs (meaning macroheterogeneity is relatively low). N-Glycosites often occur with low peptide-level valency, as well, meaning that N-glycopeptides often have only one or two modified N-glycosites. This is biased, of course, by our ability to sequence singly modified N-glycopeptides more readily than multiply-modified species, but even so, many peptides contain only one N-glycan sequon (N-X-S/T) to consider. Mucin-type O-glycosylation also exhibits high site-specific microheterogeneity, in addition to a more pronounced variation in macroheterogeneity (*i.e.*, occupancy) owing to a family of ∼20 polypeptide N-acetylgalactosaminyl transferases that differ in expression levels and protein substrate preferences across tissues and physiological conditions (38). Perhaps most challenging to MS-based analysis, mucin-type O-glycosites generally present with a high degree of peptide-level valency in combination with high microheterogeneity, meaning that several O-glycosites occur in close proximity (often on the same glycopeptide) but with potentially different glycans at each site. O-GlcNAc differs substantially from O-GalNAc in its microheterogeneity (because O-GlcNAc modifications are generally the single monosaccharide), but occupancy and peptide-level valency considerations remain similar to O-GalNAc classes.

MS-based approaches can capture these degrees of heterogeneity among all classes of glycosylation, but the presence of glycoforms and the chemistry of glycoproteins relative to nonmodified proteins necessitate enrichment, *i.e.*, the separation of glycoconjugates from nonglycosylated background species, prior to MS analysis. Glycoforms split the signal for molecules with a single glycosite into many different channels, a problem that is further exacerbated for molecules with multiple glycosites. Nonglycosylated sequences do not suffer equivalently from this signal dilution issue, reducing the abundance of individual glycoforms relative to nonmodified species. In other words, a glycosite modified with five different glycans will generate glycopeptides each with one-fifth the abundance of total signal occupied by that glycosite, assuming each glycan modifies the site with equal abundance. Nonmodified peptides from that same protein then appear 5-fold more abundant than each glycopeptide, despite their identical biological source. Thus, separation of glycosylated species from nonmodified species greatly improves sensitivity. Furthermore, hydrophobic molecules tend to ionize more efficiently in electrospray ionization, leading to signal suppression of glycosylated sequences that harbor hydrophilic glycan moieties (39, 40, 41). Glycan chemistry can further hamper ionization efficiency with the presence of acidic or negatively charged glycans because the majority of MS-based (glyco)proteomics relies on cation analysis in the positive mode. As such, enrichment of glycosylated peptides and proteins has become commonplace in glycoproteomic experiments, even when analyzing relatively simple mixtures.

**Fig. 2:**
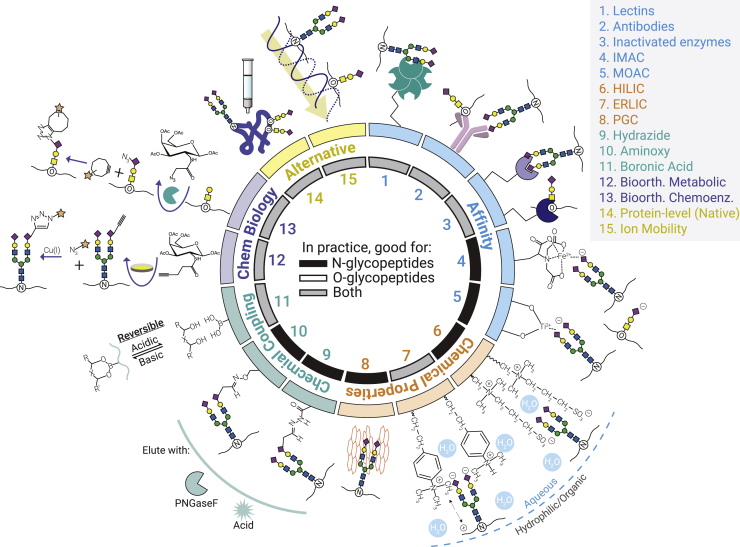
**A summary of glycosylation enrichment strategies**. Glycosylation is a heterogeneous modification that cannot be fully enriched with a single approach. Glycopeptides can be enriched along multiple axes, including affinity for glycans *via* glycan-binding proteins or charged moieties, chemical properties such as charge or hydrophilicity, chemical coupling of glycans to stationary phases, and incorporation of bioorthogonal handles through various chemical biology approaches. Alternative approaches, like native mass spectrometry (intact protein-level analysis) and gas-phase separations *via* ion mobility spectrometry are also gaining popularity in glycoproteomics. Here, the inside circle shows the suitability of each approach for N-glycopeptides (*black*), O-glycopeptides (*white*), or both classes (*gray*). The lack of white labels shows that no broad category of method is exclusively suited for O-glycopeptides, although specific implementations may be O-glycosylation oriented (*i.e.*, certain metabolic sugar analogs). Details of the figure include: antibody recognition and inactive glycosidase/glycoprotease recognition of glycan or [glycan + peptide] epitopes, an example of ZIC-HILIC stationary phase, SAX and WAX resin examples for ERLIC (left and right structures, respectively), CuAAC using an alkynyl sugar analog and an azido probe for bioorthogonal metabolic labeling, and SPAAC using an azido sugar analog and a cyclooctyne probe for bioorthogonal chemoenzymatic labeling. CuAAC, copper-catalyzed azide-alkyne cycloadditions; ERLIC, electrostatic repulsion-hydrophilic interaction chromatography; SAX, strong anion exchange; SPAAC, strain-promoted azide-alkyne cycloaddition; WAX, weak anion exchange; ZIC-HILIC, zwitterionic hydrophilic interaction chromatography.

**Fig. 3:**
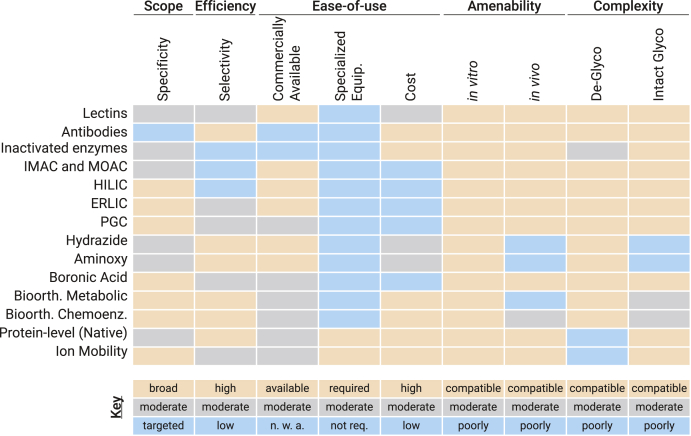
**Considerations when choosing a glycopeptide enrichment approach**. Success of various glycoproteomic enrichment strategies depends on several experimental conditions. Selection of the appropriate enrichment tool must be evaluated based on criteria that include scope of the experiment (broad *versus* targeted glycan class specificity), enrichment efficiency (*i.e.*, selectivity), the ease of use and implementation, amenability to various biological systems, and the complexity of the analytes to be analyzed (deglycopeptides *versus* intact glycopeptides). The abbreviation “n. w. a.” stands for “not widely available,” and “poorly” is short for “poorly suited.”

A number of established enrichment approaches leverage specific properties imparted by glycan moieties. Growing interest in glycobiology, coupled with recent improvements in MS-based instrumentation and software, has also rapidly expanded the collection of enrichment techniques used in glycoproteomic experiments. Here we review principles of common enrichment strategies and discuss their use in glycoproteomic experiments, with an attention paid mainly on efforts to characterize glycopeptides (rather than glycoproteins). We focus on research from the past 5 years in particular, as well. Figure 2 summarizes strategies discussed herein with the mode of enrichment depicted, and Figure 3 provides important considerations that govern what enrichment methods may be best suited for a given application. It is important to emphasize early and often that a universal enrichment strategy does not exist for glycoproteomics. Instead, different approaches can be tailored to glycan classes of interest, meaning researchers must be pragmatic when designing experiments. Our goal here is to provide perspective on which methods are best suited for different needs. We also briefly discuss how these enrichment strategies fit into broader efforts to improve glycoproteome analysis, from sample preparation through data analysis. We direct readers to prior reviews on glycoproteomic enrichment strategies for more historical perspectives (42, 43, 44, 45, 46, 47, 48, 49, 50), and we recommend several reviews that focus on N- or O-glycosylation classes more specifically (51, 52, 53, 54, 55, 56, 57, 58, 59). We also recognize recent reviews that more categorically address glycopeptide fragmentation methods (60, 61, 62), quantitative strategies for glycoproteomics (63, 64), and informatics tools (65, 66, 67, 68, 69), all of which make glycoproteomics an exciting and burgeoning field (70, 71, 72, 73).

## Endo- and Exoglycosidases

Complex pathways of glycosyltransferases and glycosidases govern the compositions and structures of glycans. Glycosidases, the enzymes that hydrolyze sugar chains, have proven particularly useful as analytical reagents to aid in glycoproteome characterization (74, 75). Although many glycosidases exist, they can be broadly grouped into endoglycosidases, which release glycans from glycoproteins, and exoglycosidases, which release monosaccharides from the nonreducing termini of glycans (*i.e.*, they trim glycan structures but leave them still attached to proteins). The actions and specificities of glycosidases warrant reviews of their own (75, 76, 77, 78, 79), but understanding their utility in glycoproteomics is crucial (80). The most widely used glycosidase is PNGaseF, an endoglycosidase that cleaves between the innermost GlcNAc moiety of N-glycans and the asparagine residue to which they are attached (81). High-mannose, complex, and hybrid N-glycans are released by PNGaseF, with some inhibition when glycans contain a core α1,3 fucose (a linkage not found in vertebrates and primarily an insect and plant modification). The PNGaseF cleavage event leaves a deamidated asparagine “scar” at the former N-glycosite that can be detected with high-resolution MS and/or the use of heavy water during the deglycosylation reaction. PNGaseF is generally regarded as the “universal endo-N-glycosidase,” even with its few caveats (82), but other endo-N-glycosidases are used in glycoproteomics as well (*e.g.*, EndoH and PNGaseA) (81).

Much of the first wave of MS-based glycoproteomics relied on the release of N-glycans with PNGaseF prior to MS analysis, so-called deglycoproteomics glycoproteomics (51). Here, glycopeptides remain intact through the enrichment stage, but glycans are then removed to make the analytes “deglycopeptides” rather than intact glycopeptides, simplifying LC-MS/MS data acquisition and subsequent informatics requirements. Deglycoproteomic experiments laid the foundation upon which modern glycoproteomics is built, helping to define the scope and relevance of the glycoproteome (83, 84, 85, 86), and they still serve as a cornerstone of glycoproteomics technology. These methods are largely limited to N-glycoproteome characterization, however, and remove valuable information about glycosite heterogeneity. They can also introduce some artifacts in glycosite assignment (87), and nonenzymatic readdition of N-glycans following PNGaseF treatment has even been observed (88). As such, a considerable portion of the current glycoproteomic method development focuses on intact glycopeptide analysis rather than deglycopeptides. We discuss the utility of both from the perspective enrichment strategies throughout.

Furthermore, a universal endo-O-glycosidase has not yet been described, although several enzymes can remove O-glycans from peptides following trimming of O-glycans by other exoglycosidases. Alternatively, deglycosylation *via* chemical beta-elimination approaches allow mapping of formerly modified glycosites (89, 90); however, these methods do not reveal the type of beta-eliminated group (*e.g.*, glycan, phosphate) that was present without further experimentation, and they can introduce artifacts from peptide hydrolysis during the beta-elimination process. As such, O-glycoproteomics is more challenging if for nothing else than the preclusion of a facile deglycoproteomics strategy. Characterization of O-glycosites and O-glycans also faces several more challenges discussed below, especially when N-glycans are also present in a sample. Because (the majority of) N-glycans can be easily removed with endoglycosidases, most O-glycoproteomics experiments incorporate PNGaseF treatment prior to glycopeptide enrichment and/or elution steps. This not only improves enrichment efficiencies for the often-smaller O-glycans but also facilitates postacquisition data interpretation.

Glycosidases are also essential to glycomics, or the characterization of free oligosaccharides, a complementary and extremely valuable field of analytical glycobiology that is inherently intertwined with glycoproteomics. Several excellent reviews describe fundamental concepts, challenges, and advances in glycomics, which we have space to only tangentially discuss in this work (91, 92, 93, 94, 95). Finally, we note that glycosylation heterogeneity described throughout this review, which brings many challenges to glycoproteomics, can be addressed to various degrees by the use of endo- and exoglycosidases to either remove or simplify glycans, respectively. Even so, this reduces the biological context captured in an experiment. Defining glycosylation heterogeneity within a system of interest is often the very point of the glycoproteomic analyses, mandating strategies to characterize intact glycopeptides with biologically relevant glycans attached to their endogenous sites. However, deglycoproteomics may remain the most advantageous approach when under extremely sample limited conditions, where heterogeneous glycopeptide signal can be condensed into fewer channels *via* deglycopeptides.

## Lectins and Affinity Chromatographies

Affinity chromatography is the broad term for leveraging specific biochemical interactions of analytes with immobilized ligands to enrich species of interest from background matrices, and it has a long history of use in concert with MS-based proteomics and related approaches (96, 97). In glycoproteomics, lectins have been the affinity chromatography approach of choice for decades because of their recognition of carbohydrates (98, 99, 100). These carbohydrate-binding proteins are present in all living organisms and can discriminate between glycan structures with varying degrees of specificity for both glycans and the substrates they modify. Hundreds of lectins, typically bound to support materials such as agarose or POROS (poly(styrene-divinylbenzene)), have been used in glycoproteomic experiments, with a majority of widely used lectins like concanavalin A (ConA), wheat germ agglutinin (WGA), *Sambucus nigra*, ricinus communis agglutinin (RCA), and jacalin coming from plants. Notable examples of mammalian lectins that will not receive due attention here include galectins (which recognize β-galactoside-containing glycans) (101) and sialic acid–binding immunoglobulin-type lectins (Siglecs) (102), which can be used in pulldown assays to define specific ligands.

Lectin specificities can be leveraged to enrich desired glycoforms for glycoproteomic analyses. For example, ConA is known to favor α-mannose-containing glycans such as those found in many N-glycans. Native WGA recognizes GlcNAc and sialic acid residues present in N-glycans and O-GlcNAc glycosylation; however, succinylated WGA improves the specificity for beta-GlcNAc. The most commonly observed specificities across lectins are for galactose, GlcNAc, glucose, mannose, fucose, GalNAc, and Neu5Ac. The use of lectins for enrichment is generally referred to as lectin affinity chromatography (LAC), and often combinations of multiple lectins are used to enrich various classes of glycopeptides from the same sample, *i.e.*, multilectin affinity chromatography (M-LAC) (103).

LAC has been successfully used for a large number of both N- and O-glycoproteomic experiments. WGA is among the more widely used lectins because of its recognition of GlcNAc residues that are found in many different glycan types. Indeed, many efforts that target O-GlcNAc glycosylation use WGA (104, 105, 106, 107, 108, 109). In an interesting set of studies, Trinidad, Burlingame, *et al.* (110) used microbore columns packed with WGA conjugated to POROS beads to investigate O-GlcNAc cross talk with phosphorylation in murine synapses. They followed this with work using WGA enrichment to profile both N- and O-glycopeptides from murine synaptosomes, one of the most comprehensive glycosylation profiling efforts to date (111). This experiment, which captured >2500 unique N- and O-linked glycopeptides, demonstrated the broad range of glycan classes WGA can enrich, including both intracellular O-GlcNAc and endoplasmic reticulum and Golgi-derived N- and O-glycopeptides. Of interest, WGA columns have been continued to be used by Medzihradszky and co-workers to characterize several glycopeptide classes, including sialylated N- and O-glycopeptides (112), mucin-type O-glycopeptides (113), and O-glycopeptides with extended and acetylated sialic acid structures (114, 115).

WGA is often used together with other lectins in M-LAC approaches, where combinations with ConA, jacalin, and RCA lectins are common (116, 117, 118, 119, 120, 121). Zielinska *et al.* (83) used ConA, WGA, and RCA lectins for their N-glyco-FASP method that mapped >6300 N-glycosites on >2300 N-glycoproteins from several mouse tissues in deglycoproteomic experiments. Jacalin has also proven useful for O-glycopeptide enrichment, both alone (122) and in combination with WGA (123) or ConA (124). Jacalin generally enriches for core-1 O-glycans with and without sialylation, although it also binds mannose residues. Alternatively, M-LAC can also be accomplished with lectins that are not used as commonly, such as King *et al.* (125) who used *vicia villosa* agglutinin (Tn-antigen) and peanut agglutinin (T-antigen, *i.e.*, the nonextended core-1 O-glycan) to profile native O-glycosites in platelets, plasma, and endothelial cells. Core-1 and core-2 O-glycans are found on glycoproteins found on many different cell types, whereas core-3 and core-4 O-glycans are more restricted to glycoproteins in the gastrointestinal and bronchial tissues (18). As such, lectins targeting core-1 and core-2 O-glycan enrichment are more widely used for general glycoproteome analysis. In another example of lesser-known lectins in M-LAC, Totten *et al.* (126, 127) combined *Aleuria aurantia* lectin (AAL), *Phaseolus vulgaris* leucoagglutinin, and *P. vulgaris* erythroagglutinin to separate core-fucosylated and highly branched glycans. Even still, new lectins with specific binding capacities are being discovered, such as SL2-1 from *Streptomyces rapamycinicus* that binds α1-6 fucosylated N-glycans but not core α1-3 fucosylated N-glycans (128). Thus, the combinatorial space of M-LAC is vast, allowing flexibility for researchers to tailor enrichments to their experimental questions with some degree of specificity.

Even with the examples described above, one challenge with lectins is defining their specificities. Although it is generally accepted that some lectins have broader binding specificities compared with others, establishing these criteria can require meticulous, dedicated characterization of each lectin of interest (129, 130, 131). Many structural characteristics combine to define glycan recognition, including networks of hydrogen bonds, van der Waals contacts, ionic bonds, and bridging interactions of water molecules and bivalent cations (132, 133). Furthermore, many lectins exist in oligomeric states that harbor several carbohydrate-binding sites. Avidity through multiple binding events (*i.e.*, multivalency), combined with nonspecific interactions of lectin domains that are not involved with glycan binding (enrichment efficiencies of ∼50% glycopeptides relative to nonglycosylated species are common) can complicate the specificities that are empirically observed.

The number of lectins available for glycoproteomic applications can be daunting, which has led to the development of databases to catalog their specificities, structures, and sources. The Glyco3D portal contains three-dimensional structures and information (134), LectinDB provides information mainly about plant lectins (135), and the Lectin Frontier Database documents lectin specificities (136). A recent concerted effort called UniLectin3D has connected many different axes of glyco(bio)informatic data, cross-referencing the aforementioned databases with other repositories, structural biology databanks, and other curated information (132). Resources like these make selection of appropriate LAC conditions a feasible task for most glycoproteomic needs.

As glycoproteomics shifts toward broad characterization efforts that aim to capture large swaths of the glycoproteome, LAC has somewhat fallen out of vogue in favor of other enrichment techniques (see below). In principle, M-LAC approaches could be designed to capture the majority of heterogeneous structures present in a glycoproteome, but the scale needed to accomplish such a task is prohibitive. That said, experiments that seek to characterize glycoproteins and glycoproteomes with defined glycan structures highlight how valuable LAC remains to glycoproteomics. Perhaps there is no better example of this than with the SimpleCell regime described by Clausen and co-workers (137). The genome editing approach to modify glycosylation pathways has enabled tunable glycoproteomes to be expressed on cells in a modular manner (11, 138). With SimpleCell, the COSMC chaperone gene for C1GALT1 is deleted, eliminating elongation of mucin-type O-glycosylation and leaving either a single GalNAc or an STn O-glycan at each glycosite (139, 140, 141, 142). This enables enrichment by lectins like *Vicia villosa* agglutinin, which has a strong preference for single GalNAc moieties on serine and threonine residues. Thus, only one or a small subset of lectins are required to enrich the entire genetically truncated O-glycoproteome that has limited O-glycan structures. This idea can be extended to O-mannose glycans, as well, enabling the enrichment of genetically truncated O-mannose glycoproteome with lectins like ConA (143, 144). The benefits of the limited lectin space required for enrichment is also relevant when using exoglycosidases rather than genomic approaches to simplify O-glycans, which can then be enriched with lectins that recognize the truncated structures (145, 146, 147).

Beyond naturally occurring lectins, there are also modified glycosidases acting as lectins reported in the literature. One example includes an O-GlcNAcase mutant that binds O-GlcNAc sites with nanomolar affinity (148). Another example includes the engineered α2,3-specific and pan-specific sialidases developed by Lectenz that have affinity and specificity for sialic acid (149, 150). For N-glycoprotein and N-glycopeptide enrichment in particular, Fbs1 carbohydrate-binding protein (which functions in the ubiquitin degradation system) has been engineered with high selectivity for N-glycan pentasaccharide core (Fig. 1, paucimannose structure) in a wide range of N-glycan classes (151). In addition to these approaches, nanoparticles with lectin functionalities have also been made to enable glycopeptide enrichment (152, 153), although this has not become widespread. Regardless of the popularity of LAC for MS-based glycoproteomics and related technologies in the coming years, lectins continue to play important roles in a number of other assays used in glycobiology research, as well, including lectin histochemistry, lectin blotting, enzyme-linked lectin assays, and lectin arrays (154).

Antibodies toward glycans and glycosylated proteins can also be used for enrichment (155). O-GlcNAc and O-GalNAc (specifically, the Tn-antigen on MUC1) antibodies have been reported (156, 157, 158, 159, 160, 161, 162), although they have limited use in glycoproteomics thus far. Antibodies have also been used for immunoprecipitation of fucosylated N-glycosylation epitopes (163, 164), but the targeted nature of these enrichments limits their broad use for either N- or O-glycoproteomics. Cummings and co-workers have developed smart antiglycan reagents, which are generated by immunizing lampreys (*Petromyzon marinus*) with glycoconjugates to induce secretion of variable lymphocyte receptors as antibodies toward specific glycan epitopes (165, 166). These may prove valuable for MS-based glycoproteomics but so far have mainly been used in microarray formats.

### Immobilized Metal Affinity Chromatography and Metal Oxide Affinity Chromatography

Outside of LAC, immobilized metal affinity chromatography (IMAC) and metal oxide affinity chromatography (MOAC) have gained popularity in enriching negatively charged (*i.e.*, sialylated) glycans. Much of the development in IMAC and MOAC methodology grew from efforts to improve phosphoproteomic workflows (167, 168). IMAC is accomplished by chelating transition metal cations (Fe^3+^, Ga^3+^, Ti^4+^, Zr^4+^, etc.) onto immobilized substrates, whereas MOAC uses transition metals in metal oxide matrices (most commonly titanium dioxide, TiOx). Both techniques leverage the affinity of deprotonated oxygens in phosphate moieties in phosphoryl groups, but this affinity is not exclusive to phosphopeptides. Thus, deprotonated carboxylic acid groups, such as those on aspartic and glutamic acid side chains, are also enriched with IMAC and MOAC. Similarly, these enrichment modalities select for carboxylic acid anions and hydroxyl groups in sialic acids to enable the enrichment of sialylated glycopeptides, in addition to glycopeptides with phosphorylated glycans (*e.g.*, mannose-6-phosphate, M6P).

In 2007, Larsen *et al.* reported enrichment of sialylated glycopeptides from human plasma and saliva using TiOx enrichment with acidic buffer conditions (169, 170, 171). They used alkaline phosphatase to avoid copurification of phosphopeptides in this first iteration, but they later utilized TiOx in combination with sequential IMAC elutions with acidic and basic buffers for simultaneous phospho- and glycopeptide enrichment (172). Here, IMAC enrichment is followed by TiOx enrichment to separate multiphosphorylated peptides from monophosphorylated and glycopeptides. These methods have been used to characterize glycoproteomes (and sometimes phosphoproteomes) of stem cell differentiation (173), Alzheimer’s-related neuroproteome changes (174), and stimulated rat neurons, where site-specific glycosylation can change after only seconds of depolarization (175). Of importance, others have shown that sialylated N-glycopeptides can be enriched not only by TiOx but also by Fe^3+^-IMAC (176) and Ti^4+^-IMAC (177). Coenrichment of phosphopeptides and glycopeptides can also be achieved with hydrophilic interaction chromatography (HILIC, see below) (178, 179) and nanomaterials functionalized with HILIC matrices, metal ions like Ti^4+^ or Ga^3+^, or both (180, 181, 182).

The above-mentioned experiments relied largely on PNGaseF for N-glycan removal following enrichment, enabling characterization of formerly sialylated deglycopeptides. Several groups have extended these IMAC and MOAC enrichment methods for characterization of intact glycopeptides. Glover *et al.* (183) showed that intact N-glycopeptides with both sialic acid- and M6P-containing glycans can be identified from Fe^3+^-IMAC enrichments, ultimately characterizing ∼4000 phosphopeptides and ∼1000 N-glycopeptides simultaneously. Subsequently, Huang *et al.* (184) showed that Ti^4+^-IMAC can also be used for M6P-glycopeptide enrichments. Around the same time, Hu *et al.* (185) showed that thousands of previously unidentified intact sialylated N-glycopeptides could be found in published IMAC-enriched phosphoproteomic data sets. Cho *et al.* (186) found that coenrichment of phosphopeptides and glycopeptides during IMAC enrichment was inevitable no matter what buffer pH conditions were used. They showed that a subsequent enrichment of IMAC elutions with mixed-mode HILIC separations (discussed in detail below) could largely separate phosphopeptide and glycopeptide populations, enabling identification of ∼18,000 phosphopeptides and ∼3500 intact glycopeptides when using additional offline fractionation prior to MS analysis (186). Exciting recent studies from multiple groups have shown that these combinatorial enrichments can be used to characterized phospho- and glycoproteomes of patient-derived xenograft tumor tissues and extracellular vesicles from human plasma and urine (187, 188, 189).

Of interest, differing reports of glycopeptide *versus* phosphopeptide selectivity in IMAC and MOAC methods are still emerging. Although Fe^3+^-IMAC is known to enrich sialylated glycopeptides as described above, Caval *et al.* (190) reported that a column-based Fe^3+^-IMAC matrix (opposed to magnetic beads, etc.) was selective for M6P-containing glycans with little coenrichment of sialylated species. They compared this enrichment to Ti^4+^-IMAC and TiOx enrichments to show this selectivity was specific to their Fe^3+^-IMAC conditions, indicating there is still much to explore as to why some conditions promote coenrichment of sialylated glycopeptides while others seem more selective for phosphorylated species. As IMAC and MOAC matrices with different metal ions are developed and applied to phosphoproteome characterization (191), it will be important to characterize which translate best for characterization of various subpopulations of the glycoproteome. In addition, most IMAC and MOAC enrichment studies have focused on N-glycopeptides, so future studies will need to explore its utility for enriching sialylated, sulfated, and otherwise anionic O-glycopeptides, presumably following enzymatic removal of confounding N-glycans. Outside of direct coenrichment of phospho- and glycosylated peptides, IMAC and MOAC enrichments of phosphopeptides also remain relevant enrichment tools for investigating cross talk of phosphorylation and O-GlcNAcylation (192). In all, the IMAC and MOAC methods may not be well suited for sample-limited situations, owing to the coenrichment of phosphopeptides and possible requirements of multiple handling steps. Informatic challenges of searching for cophosphorylated and glycosylated peptides must also be considered. That said, coenrichment could enable low sample loading amounts if specificities between phospho- and glycopeptides can be properly tuned.

## Hydrophilic Interaction Chromatography

HILIC is an indispensable tool for glycoproteome enrichment and characterization. Glycopeptide enrichments *via* HILIC have been long established and continue to remain popular across the field (193, 194, 195, 196). HILIC has utility both as an enrichment mode prior to reversed-phase LC-MS/MS analysis of glycopeptides and as the chromatography mode used in liquid chromatography directly coupled to MS analysis. Our focus here is primarily on HILIC for enrichment prior to data acquisition rather than an online separation during LC-MS/MS, but we direct readers to several excellent resources for HILIC analyses for both glycopeptides and glycoproteins (194, 195, 196, 197, 198, 199, 200, 201). As with many of the enrichment approaches in this review, the literature of HILIC applications in glycoproteomics is vast and impossible to cover exhaustively. Instead, we aim to cover the basic principles and highlight demonstrative examples that highlight its utility as well as limitations for glycoproteome characterization. We also note that HILIC enrichment can be coupled with deglycoproteomic analysis, but the majority of recent studies using HILIC and its derivatives have focused on intact glycopeptide characterization.

HILIC enriches glycopeptides *via* the hydrophilic properties imparted by the glycan, although hydrophilic nonglycosylated peptides can also be coenriched (202). HILIC employs semiaqueous mobile phases to create a “water-enriched” layer within a hydrophilic stationary phase. Separation/enrichment is achieved as hydrophilic glycopeptides partition from organic loading buffers into this hydrophilic environment (203, 204). The primary function of the stationary phase is to bind water, and there are many HILIC stationary phases that can be suitable for glycopeptide enrichment (205). The most widely used are zwitterionic HILIC matrices, where electrostatic forces of the charged stationary phase are (partially) counterbalanced by the proximity of opposite charges. This permanent zwitterion reduces secondary electrostatic interactions that can significantly alter HILIC retention on charged stationary phases but enables higher selectivity than neutral HILIC matrices (86).

HILIC mobile phases are an integral part of the stationary phase (*i.e.*, forming the “water layer”), meaning the water fraction of the mobile phase greatly dictates retention. Generally, at least 3% to 5% water is required in any HILIC buffer to retain sufficient hydration of the resin. Elution of glycopeptides is typically achieved with buffers with a water fraction of ∼50% to 60%, whereas higher concentrations of organic solvents increase retention of glycopeptides on the column. Several water-miscible organic solvents are compatible with HILIC separations, but the choice of organic solvent has significant effects on glycopeptide enrichment outcomes.

Alagesan *et al.* (206) evaluated the effects of organic mobile phases for HILIC enrichment while also comparing solid-phase extraction (SPE) and Drop-HILIC (*i.e.*, adding HILIC resin to resuspended peptide mixtures) formats. Their results show that many nuances exist in HILIC enrichment, but generally acetonitrile is the most favorable organic solvent to use (*i.e.*, the fewest number of coenriched nonmodified peptides). Isopropyl alcohol and ethanol can be more beneficial for larger, more hydrophobic glycopeptides, but methanol was poorly suited for HILIC enrichment, presumably from disruption of hydrophilic partitioning *via* hydrogen bonding. In addition, they comment on the comprised enrichment efficiency of SPE HILIC in the presence of salts that are common in reduction/alkylation and proteolysis steps to prepare glycopeptides prior to enrichment. Desalting with C18 material is a nearly ubiquitous step in proteomic workflows that can alleviate this problem, but highly hydrophilic glycopeptides can be lost in this step (207). They recommend a chloroform–methanol precipitation to remove salt instead, although precipitation steps can also be prone to some sample loss. In addition, formic acid is widely used in HILIC enrichment protocols to reduce the coenrichment of hydrophilic nonglycosylated peptides, which can introduce artifacts like glycan formylation (208). Thus, even with the success HILIC enrichments provide for glycoproteomics, several factors could yet be optimized.

HILIC enrichments have been used to enrich glycopeptides from a wide range of biological matrices, including human biofluids (*e.g.*, plasma, serum, and milk) (209, 210, 211, 212, 213, 214), cancer systems (215, 216, 217, 218, 219, 220, 221), other human model systems (222, 223), murine tissues (178, 224, 225, 226, 227), pathogens (228, 229, 230, 231), and plants (232). HILIC has been used both as stand-alone and in combination with IMAC and MOAC to enrich phospho- and glycopeptides, too, as mentioned above. Even though HILIC is often thought to be suitable for generic glycoproteome interrogations (233), it is generally best suited for glycopeptides with relatively high glycan-to-peptide ratios by mass, *i.e.*, glycosylated species with large, branching glycans and/or multiply-glycosylated peptides (202). This biases many HILIC applications to N-glycoproteomics, with a disadvantage for paucimannose N-glycans and O-glycopeptides that tend to have smaller glycans (17, 52, 234, 235). Several studies have successfully leveraged HILIC for O-glycopeptide characterization, including human serum and urine (236, 237, 238, 239), *Burkholderia* and *Acinetobacter* bacteria (230, 231), and simple mixtures of O-GalNAc peptides (240, 241). Even so, HILIC may not be the most suitable first choice of enrichment method for studies aiming to characterize the O-glycoproteome.

Several groups have described methods to synthesize or fabricate HILIC resins to continue expanding HILIC capabilities. A common property achieved by these materials is hydrophilicity, although the avenues to achieve such properties differ. Materials include cobalt sulfide nanoleaves (242), covalent/metal organic framework approaches (181, 243, 244, 245, 246), carbon microspheres (247), and polymeric monoliths functionalized with piperazine (248), among others. As described above, some of these materials can have bifunctional properties to enable HILIC-based glycopeptide enrichment and metal ion–based phosphopeptide enrichment (181, 182, 249).

### Electrostatic Repulsion–Hydrophilic Interaction Chromatography

Mixed-mode HILIC approaches have also gained popularity in recent years. In mixed-mode approaches, the hydrophilic stationary phase has an electrostatic charge (rather than the zwitterionic balance), which superimposes electrostatic interactions with analytes on top of hydrophilic partitioning (250). In these so-called electrostatic repulsion–hydrophilic interaction chromatography (ERLIC) methods, a charged ion exchange column is used with HILIC mobile phases (*i.e.*, high concentrations of organic solvents). The goal is to match or complement the polarity of the ion exchange column with the charge of analytes. Under normal aqueous conditions, electrostatic repulsion between the stationary phase and the analytes would exclude analytes from binding/interacting. However, high organic solvents drive hydrophilic interactions between the polar analytes and the water layer of the stationary phase, and analytes can be retained despite the electrostatic repulsion. This balance can then be modulated *via* pH to alter the electrostatic charge of the analytes (251), which can be used to select for specific functional groups. For glycopeptides, ERLIC stationary phases are usually anion exchange (AX) resins (which have a positive charge), and charges of basic amino groups on peptide backbones and acidic sialic acids on glycans are controlled with buffer pH. This extra degree of tunability gives ERLIC some advantages over zwitterionic HILIC, especially for enriching charged (*e.g.*, sialylated, sulfated) glycans that will have electrostatic attraction to AX stationary phases.

AX resins can be made as weak anion exchange, strong anion exchange (SAX), or mixed-mode strong anion exchange (MAX) based on the functional groups used. MAX resins have attenuated anion affinity, *e.g.*, *via* tertiary amines, that can have a broader selectivity for acids than the strong acid preference of SAX resins. The properties of the AX resin can affect glycopeptide retention, so glycoproteomic ERLIC methods generally include a title descriptive of the resin used. Following a study from Cao *et al.* (252) describing SAX-ERLIC for small-scale glycopeptide enrichment, Totten *et al.* (253) described SAX-ERLIC enrichment for N-glycopeptides from human plasma, benchmarking it against M-LAC and HILIC enrichment. The SAX-ERLIC approach generated the most extensive glycopeptide data of the three and yielded over four times as many unique glycopeptides and half as many nonglycosylated peptides as the HILIC method. Even with the selectivity provided, SAX-ERLIC still had an enrichment efficiency of ∼50% (ratio of glycopeptide identifications to total identifications). Zacharias *et al.* (254) compared HILIC and ERLIC, too, although they achieved slightly higher identifications with HILIC. They also reported the two methods to be somewhat complementary and performed sequential HILIC-ERLIC enrichments that provide new identifications not found in HILIC-only analyses (254). Cui *et al.* (255) recently expanded SAX-ERLIC investigations with a detailed study into the “sweet spot” of ERLIC mobile phases that would enable the most selective N-glycopeptide enrichment. They reported that 80% acetonitrile outperformed 95% acetonitrile buffers for hydrophilic partitioning in ERLIC methods (this matches previous HILIC reports (206)), improving the specificity for modified *versus* nonmodified peptides. They also saw that trifluoroacetic acid performed better as an ion pairing agent than formic acid for N-glycopeptide enrichment and that elution of N-glycopeptides is mainly governed *via* disruption of hydrophilic interactions through increased water content (rather than coulombic interactions with pH/salts).

Yang *et al.* (256) reported large-scale N-glycoproteomics with MAX-ERLIC enrichments, identifying 10,338 unique N-glycopeptides that correspond to 1163 N-glycosites on 530 glycoproteins. This, and other studies mentioned above, indicates that ERLIC-based approaches are adept for generic N-glycoproteome interrogations, similar to HILIC methods. That said, ERLIC methods likely have a bias toward charged, sialylated glycopeptide enrichment. MAX methods used by the Zhang group (186) were also referenced above, as they enabled simultaneous profiling of both phospho- and glycopeptides. Similarly, Cui *et al.* (255) showed phosphopeptide coenrichment with SAX-ERLIC. Of importance, this indicates that, although ERLIC-based methods may provide broad N-glycoproteome enrichment, N-glycopeptides are not the exclusive species enriched in these methods, and some sensitivity issues may remain owing to lack of selectivity. Also, it is important to note that ERLIC methods can fail to properly enrich some glycan classes, such as fucosylated glycans seen in a report from Zhou *et al.* (257), showing that combinations of enrichment methods can often be beneficial.

As with the majority of enrichment methods, more data are available for ERLIC methods when characterizing N- rather than O-glycopeptides. ERLIC has the same limitations in glycan-to-peptide mass ratio considerations that HILIC methods do, although this may be overcome to some degree when seeking to enrich negatively charged glycopeptides. Some evidence of ERLIC methods working for O-glycopeptides include a 2017 study from Yang *et al.* (258), which compared HILIC and two different SAX-ERLIC methods for both N- and O-glycopeptides. They reported several important findings, including 1) improved HILIC enrichment of O-glycopeptides following PNGaseF treatment, 2) improved identification of N- and O-glycopeptides with MAX- and SAX-ERLIC, 3) improved O-glycopeptide enrichment with SAX-ERLIC compared with MAX-ERLIC, and 4) compatibility of ERLIC methods with tandem mass tag isobaric labeling. Other studies have also used ERLIC methods for O-glycopeptide enrichment with varying degrees of success (145, 259, 260). Although this is encouraging, more data are likely needed to know how generalizable ERLIC will be for characterizing the O-glycoproteome as methods improve the depth at which the O-glycoproteome can be sampled. Overall, HILIC and its derivatives like ERLIC will continue to be a mainstay in glycopeptide enrichment, and we expect them to be utilized in large-scale quantitative glycoproteomic studies for the foreseeable future.

## Porous Graphitic Carbon

Alternative chromatographic modes that enable retention of polar and hydrophilic species have always been an interest for many bioanalytical fields that traditionally rely on reversed-phase separations. Porous graphitic carbon (PGC), developed to overcome shortcomings of silica-based stationary phases, is a crystalline material composed of sheets of graphitized carbon that are held together through van der Waals interactions in a hexagonal arrangement, although successive layers are not oriented regularly (differentiating it from traditional three-dimensional graphite) (261). PGC retains polar analytes with unique behavior relative to other stationary phases, although the mechanisms of this retention are not fully understood (261, 262). Hydrophobicity and charge contribute to retention, as does molecular shape, which are partially explained through dipole–dipole interactions induced on the surface by charged analytes and electronic repartition on the graphic surface that places an electronic excess at the edges of planes within the PGC structures (261). Whatever the reasons, PGC has proven useful for enriching and separating glycans and glycopeptides (263, 264, 265, 266, 267, 268, 269, 270).

PGC can be used both in SPE formats to enrich glycopeptides and in online methods, sometimes in combination with reversed-phase separation, for LC-MS/MS. PGC-SPE formats have yet to be widely adopted for enrichment (271, 272), likely because complicated retention mechanisms make method optimization challenging and less intuitive. That said, PGC has the attractive potential of chromatographically separating isomeric glycopeptides based on their glycan structures, and several studies have utilized online PGC separations to characterize intact glycopeptides (233, 273, 274, 275, 276, 277, 278, 279, 280). One challenge is that hydrophobic glycopeptides are difficult to elute from PGC, so most studies using online PGC separations have relied on nonspecific proteases to generate small peptide backbones for glycopeptides with limited hydrophobicity. Coupling of PGC with reversed-phase separations can help (281, 282), but the use of nonspecific proteases still creates an additional layer of peptide backbone heterogeneity on top of the already challenging glycosylation heterogeneity present in the sample. Mechref and colleagues recently reported improved PGC-LC-MS characterization of N-glycopeptides from cleavage- specific protease (trypsin/LysC, GluC, and chymotrypsin) through the use of higher temperatures and basic (rather than acidic) mobile phases (283), although higher temperatures have been reported to improve glycopeptide isomer discrimination for reversed-phase separations, too (284). The potential uses of PGC for glycoproteome characterization are exciting, but it remains to be seen if the technical challenges associated with this unique stationary phase will limit its application to large-scale analyses.

## Chemical Coupling Strategies

Glycans inherently contain functional groups that can be directly targeted for enrichment (285). Historically, hydrazide chemistry has been widely used to profile sialylated glycoconjugates (286). Hydrazine groups (NH_2_-NH_2_) can selectively react with aldehydes, which do not naturally exist in glycans but can be generated by the periodate oxidation of sialic acids. A wide range of scaffolds can incorporate hydrazide functional groups that can then be leveraged to enrich glycopeptides containing oxidized sialic acids. One of the first applications of hydrazide chemistry for enrichment in glycoproteomics was by Zhang *et al.* (287), who used a solid support matrix functionalized with immobilized hydrazide groups to covalently capture sialylated glycopeptides. PNGaseF was then used to release N-glycopeptides from the solid support, enabling characterization of de-N-glycopeptides.

This approach has been expanded in a number of ways since then, with uses in both N- and O-glycoproteomics (288, 289, 290, 291, 292, 293, 294, 295, 296, 297). One recent improvement on the method, called solid phase extraction of *N*-linked glycans and glycosite-containing peptides, tethers glycopeptides to solid support through the peptide N terminus (after blocking other ε-amino groups with guanidination) rather than the glycan (298). Several other steps, including anilination of carboxylic acids, N-glycan release with PNGaseF, deglycopeptide elution *via* AspN digestion, and separate analysis of enriched intact glycopeptides, ultimately generate information about the total *N*-linked glycan, glycosite-containing peptide, and glycoprotein content of complex samples. Variations of this method has been used for a variety of applications, including characterization of atypical N-glycosites that have N-X-C motifs (299), and it has been recently reported to outperform HILIC and other solid-phase enrichment methods (300). Similar approaches have also been adopted for O-glycopeptide analysis (301, 302, 303). These methods are largely enabled by a newly described O-glycoprotease called OpeRATOR, an enzyme from the gut commensal *Akkermansia muciniphila* (304). O-Glycopeptides are released from solid support matrices by OpeRATOR cleavage, which leaves an O-glycosite at the N-terminal peptide residue (259). However, even though the N-terminal residue will be glycosylated, it cannot be assumed to be the only O-glycosite in the sequence, meaning some complications of O-glycopeptide characterization remain (259, 305).

Hydrazide chemistry has also been fundamental to developing strategies to profile glycoproteins exclusively labeled at the cell surface. Analyzing glycoproteomes from whole-cell lysate generates confounding artifacts that complicate determination of what glycoforms are biologically relevant and present on the cell surface. Several approaches have been developed to label cell-surface glycoproteins on live cells, prior to cell lysis and protein extraction (306, 307). One variation functions similarly to the approaches above: sialic acids are oxidized on live cells *via* mild periodate treatment, and aldehyde-containing sialylated glycopeptides are captured on hydrazide beads. However, elution differs in that acidic conditions are used to cleave the linkage of sialic acids to the rest of the glycan, releasing intact (yet asialylated) glycopeptides for analysis (308, 309). The benefits of intact glycopeptide analysis are important, and this opens the method to both N- and O-glycopeptide characterization, but the acidic conditions used for elution can introduce a number of artifacts. A more widely adopted approach called Cell Surface Capture instead labels oxidized sialic acids with biocytin-hydrazide, a bifunctional molecule that can generate biotinylated species (85, 310). Thus, sialylated glycopeptides derived from cell surface glycoproteins can be enriched with streptavidin and eluted *via* PNGaseF release for de-N-glycopeptide analysis. Although this method has thus far been limited to characterization of formerly N-glycosylated peptides, it has been a valuable approach for generating cell surface glycoprotein maps of common mammalian cell lines and is used for a variety of applications (311, 312, 313, 314, 315, 316, 317, 318, 319, 320, 321, 322).

Alkoxyamine (aminooxy) compounds also conjugate to aldehydes in a similar manner to hydrazide compounds, making them viable tools for glycoproteomic enrichment. That said, they are far less utilized than hydrazide compounds in glycoproteomics. A notable exception is the PAL method from Paulsen and co-workers, which functions similarly to Cell Surface Capture (323, 324). Thiazolidine chemistry was also recently used for a similar approach (325). Multifunctional probes using hydrazide and aminooxy groups have been used to target ligand–receptor interactions at the cell surface, as well (326, 327, 328, 329). These aldehyde-selective chemistries can be expanded further to target other glycan classes if aldehyde groups can be generated on specific monosaccharides. For example, this can be accomplished through enzymatic approaches that use galactose oxidase, which can selectively oxidize galactose and galactose oxidase. Several studies have used galactose oxidase for cell surface profiling *via* hydrazide- and aminooxy-based enrichment approaches (330, 331, 332, 333, 334, 335).

Monosaccharides other than sialic acids and galactose can also be targeted with these strategies, although to a limited degree. Rannes *et al.* (336) showed that directed evolution of galactose oxidase could enable oxidation of mannose and GlcNAc residues. Oxidation of the GlcNAc *trans* diol into an aldehyde can enable O-GlcNAc enrichment through hydrazide chemistry (337), although these reactions typically require high concentrations of sodium periodate and high temperatures (337). Even so, hydrazide and aminooxy chemistry methods currently bias strongly toward N-glycoproteome analysis because of the reliance on PNGaseF for elution in a majority of cases, so new strategies will need to emerge to enable similar characterization of the cell-surface O-glycoproteome. Also, sensitivity and selectivity can be influenced by biomolecules that naturally contain carbonyl groups (*e.g.*, metabolites like glycose, pyruvate) and by side reactions that occur during periodate oxidation of glycans (*e.g.*, oxidation of methionine, cysteine, and N-terminal serine and threonine residues) (338).

### Reversible Chemical Coupling

Although carbonyl-reactive probes like hydrazide and aminooxy compounds are valuable and popular tools, boronic acid–based enrichments are arguably the more versatile chemical coupling approach for glycopeptide enrichment. Boronic acids can covalently react with *cis*-diols to form five- or six-membered cyclic esters in basic conditions, and, of importance, this reaction is reversible under acidic conditions (339, 340, 341). Indeed, glycans contain *cis* 1,2 and 1,3 diols that can conjugate with boronic acid, enabling a selective but reversible covalent glycopeptide enrichment platform (342). Boronic acid–based methods have shown potential as an unbiased large-scale glycoproteomic enrichment strategy (343, 344, 345, 346, 347, 348), but glycan–boronic acid interactions are relatively weak. This presents a disadvantage for enriching many low-abundance glycopeptides that may be outcompeted during the enrichment process. Recently, several reports have shown that boronic acid–glycan interactions can be enhanced through screening of boronic acid compounds or through increasing avidity *via* dendrimeric boronic acid–functionalized materials (345, 346, 349, 350, 351).

Boronic acid–based enrichments have the potential to characterize both N- and O-glycopeptides. O-Glycopeptide enrichment with boronic acid approaches has been reported (352), although the interactions with smaller O-glycans may be weaker and thus harder to capture. We anticipate that boronic acid–based enrichments will gain popularity as novel materials continue to improve the strength of boronic acid–glycan interactions and continue to become more available. Furthermore, continued development of reversible covalent modifications, such as a recently described Schiff base hydrolysis strategy (353), will continue to enable intact glycopeptide enrichment. Also of interest are host–guest interactions, which can enable reversible capture of glycosylated species with specificities for monosaccharides, *e.g.*, N-glycolylneuraminic acid (Neu5Gc) and N-acetylneuraminic acid (Neu5Ac) sialic acids (354). Although these have limited application in glycoproteomics thus far, they represent a potentially useful platform for future enrichment modalities.

## Chemical Biology Tools

Chemical biology approaches to glycopeptide enrichment seek to add nonendogenous chemical handles to glycans that can be targeted to separate glycosylated species from nonmodified peptides with a high degree of specificity (355, 356). Often these methods use bioorthogonal reactions, *i.e.*, chemical reactions that occur inside of living systems without interfering or interacting with native biochemical processes (357). Chemical handles can be incorporated into glycans metabolically, enzymatically, or through chemical coupling strategies discussed above (*e.g.*, hydrazide chemistry) (358).

Metabolic incorporation of bioorthogonal handles into glycans can be accomplished with chemically functionalized monosaccharides. Azides are typically chosen as the bioorthogonal handle because they are small, unreactive with endogenous cellular components, stable *in vivo*, and easy to add to synthetic sugars (359, 360). Azido-sugars can then be selectively labeled through copper-catalyzed azide-alkyne cycloadditions (CuAAC, *i.e.*, “click” chemistry), strain-promoted azide-alkyne cycloaddition (SPAAC, *i.e.*, “copper-free click” chemistry), or Staudinger ligations (361, 362, 363, 364, 365, 366). Alkynes can also be used as the bioorthogonal handle for unnatural monosaccharide incorporation, which are then labeled with azide probes (357, 359, 360).

Glycan biosynthesis pathways can metabolically incorporate unnatural biosynthetic precursors to target specific forms of glycosylation. For example, analogs of ManNAc, *e.g.*, ManNAz (azide handle) and ManNAlk (alkyne handle), get exclusively incorporated as sialic acids, providing an avenue to specifically label sialylated glycoproteins (362, 367, 368). Glycoproteomic experiments have taken advantage of a wide range of azido and alkynyl monosaccharide precursors, including variants of ManNAc, GalNAc, GlcNAc, and fucose (369, 370, 371, 372, 373, 374, 375, 376, 377, 378). Although sialylated N- and O-glycans can be specifically labeled, incorporation of other sugars can be more complicated. Fucose variants can label N-glycans (either core fucose or antennary fucose) or O-fucose glycans, but incorporation of fucose derivatives can be limited or even inhibit protein fucosylation (374, 375). GalNAc analogs can be incorporated into N- and mucin-type O-glycans, but GalNAc precursors can also be epimerized into GlcNAc *via* UDP-galactose 4-epimerase (371). This leads to labeling of both GalNAc and GlcNAc moieties when GalNAc analogs are used, complicating labeling selectivity unless UDP-galactose 4-epimerase knockouts or other genetic strategies are pursued. This metabolic cross talk also affects labeling with GlcNAc variants (379, 380), which are generally used for the goal of targeted O-GlcNAc labeling but result in colabeling of GalNAc-containing sugars. That said, O-GlcNAc analogs specific to O-GlcNAc labeling have been described (381, 382, 383), enabling profiling of O-GlcNAcylated proteins without confounding GalNAc enrichment. Metabolic labeling strategies have enabled several recent glycoproteomic studies, including N-glycoproteome profiling (384, 385), O-glycoproteome profiling (386, 387, 388), N- and O-glycoproteome profiling from the same samples (389, 390, 391), cell surface glycoproteomics (392, 393, 394), O-GlcNAc-centric glycoproteomics (395, 396, 397), and even glycoproteomics in *ex vivo* cultured human tissues (398).

Chemoenzymatic labeling of glycans with bioorthogonal handles, where enzymes append functionalized monosaccharides to native glycans, is a complement to metabolic incorporation (399). Chemoenzymatic labeling of glycans for glycoproteomic enrichment has long been used to target O-GlcNAc, which generally relies on the action of β-1,4-galactosyltransferase 1 (GalT1) in transferring a galactose to terminal GlcNAc residues. GalT1 has been engineered to accept unnatural sugar analogs that can be labeled with aminooxy or click chemistry probes to enable enrichment (400, 401, 402, 403, 404, 405, 406, 407, 408, 409, 410, 411). Alternatively, unmodified galactose can be incorporated with wildtype GalT1, galactose oxidase can be used to generate an aldehyde on the appended galactose, and hydrazide chemistry can then be used to enrich chemoenzymatically modified O-GlcNAc residues (412). Sialic acids have also been targeted with *trans*-sialidase chemoenzymatic labeling strategies (413, 414, 415, 416), and recently GalNAc (*i.e.*, Tn-antigen) was profiled by adding stable isotope-labeled galactose to terminal GalNAc residues *via* glycosyltransferase C1GalT1 (417). Selective exoenzymatic labeling has also been used to label glycans with biotinylated or azido-labeled nucleotide-sugar analogs *via* ST6Gal1 and ST3Gal1 sialyltransferases, with specificity demonstrated for N- and O-glycans, respectively (418, 419). Some glycotransferases can also be used to selectively add modified monosaccharides to specific epitopes, such as the bacterial acetylgalactosaminyltransferase used by Zhu *et al.* (420) to selectively label the Neu5Ac-α2,3-Gal epitope with GalNAz for sialic acid linkage discrimination. In practice, many of the probes used to enrich labeled glycans following metabolic incorporation can also be used for enrichment for chemoenzymatically labeled glycans. Metabolic incorporation and chemoenzymatic labeling can also be used in concert to profile the cell surface glycoproteome (56).

The biggest advantage of these chemical biology tools is their selectivity, especially because enrichments *via* bioorthogonal reactions can occur under stringent conditions that minimize nonspecific interactions. They can generally be used to profile both N- and O-glycosylation, as well, depending on experimental design. Of importance, metabolic incorporation approaches are compatible with dose- and time-dependent labeling. However, these methods require judicious selection of biological systems (*e.g.*, with controlled epimerase activity) and are limited in their applicability to human tissue and biofluid samples. Furthermore, metabolic incorporation efficiency is often low, likely owing to tolerance limits of varying biosynthetic enzymes for unnatural substrates, which limits the quantitative capacity of metabolic labeling approaches. Conversely, although chemoenzymatic methods provide a high-efficiency approach to glycan labeling, they are generally only suitable for steady-state glycosylation investigations rather than dynamic regulation. Also, developing enzymes that can tolerate unnatural monosaccharide substrates can be difficult. Despite these challenges, chemical glycobiology and chemical biology for glycoproteomics are rapidly developing fields that have generated an exciting number of improvements and novel approaches, even in the past 5 years (421). Many shortcomings have the potential to be addressed and improved upon as efforts continue.

## Other Strategies

The above approaches comprise the majority of efforts in glycoproteomic enrichment methodology, but other strategies can be equally as valuable. Acetone precipitation, for example, can be tailored to selectively enrich N- and O-glycopeptides (422). More canonical protein purification strategies can also be used to isolate specific glycoproteins of interest for characterization of glycan heterogeneity. When targeting specific proteins, both top-down (endogenous, nonproteolytically digested protein) and bottom-up (proteolytically derived glycopeptides) proteomic strategies can be used in tandem to assign prevalence of various glycoforms. Native MS approaches have proven adept in these scenarios, where glycoform heterogeneity can be observed in high m/z charge state distributions of native-sprayed glycoproteins while specific glycosite assignments are provided by complementary bottom-up analysis (423, 424, 425, 426, 427, 428, 429, 430, 431, 432).

Gas-phase enrichment and separation also continue to gain traction in MS-based proteomics as a whole and have recently demonstrated value for glycoproteome characterization (433). Ion mobility spectrometry (IMS) separates ions based on their mobility in a carrier buffer gas and is generally coupled with MS detection to provide collisional cross sections of ions along with their m/z. IMS can be particularly useful for glycopeptides because various glycan structures alter conformations of the proteins and peptides they modify, thereby changing their mobility in IMS and enabling gas-phase separation (434). There are many varieties of IMS that have been used in glycoproteomics (435), and IMS has been used to separate structural isomers of N-glycopeptides (436, 437, 438, 439, 440), isomeric O-GlcNAc and O-GalNAc glycopeptides (441, 442, 443, 444, 445), O-glycoforms of glycoproteins (446), and glycoforms with different sialic linkages (447, 448).

Of interest, Scott and co-workers recently showed that field asymmetric waveform IMS gas-phase fractionation can enrich short aliphatic glycopeptides from complex mixtures, which are often unseen in HILIC enrichments (234). The field asymmetric waveform IMS source they used enables compensation voltage ramping within the same experiment (449), which provides flexibility to improve access to both N- and O-glycopeptides. Although the method is not IMS based, Alagesan and Kolarich also recently showed that glycopeptide enrichment may not be necessary prior to LC-MS/MS when using dopant solvents in electrospray ionization sweep gases (which are used to support liquid desolvation and focus the Taylor cone) (41). Acetonitrile and acetone increased the signal intensities of synthetic and proteolytically derived IgG glycopeptides by 5- and 2-fold, respectively. These studies are new and require further investigation, but they represent intriguing opportunities to continue to improve sensitivity in glycoproteomic experiments both with and without glycopeptide enrichment prior to MS analysis.

## Related Developments in Glycoproteomics

Glycopeptide enrichment is a crucial component to any glycoproteomics workflow, but several considerations in sample preparation, data acquisition, and data analysis also determine experimental outcomes. Perhaps most fundamental is the way proteins are extracted from biological systems for further processing. Proteomic workflows can bias against membrane proteins and highly hydrophilic or hydrophobic subsets of the proteome (450). Several approaches have been developed to address these methodological blind spots, and they should benefit glycoproteomic methods, too. Specifically, sample preparation protocols that permit the use of detergents during protein isolation should improve characterization of membrane-bound glycoproteins, *i.e.*, a substantial portion of the glycoproteome (451, 452, 453, 454, 455, 456, 457, 458). Automated sample preparation protocols also promise to streamline glycoproteomics in concert with other “omics,” including glycomics and deglycoproteomics (459). Furthermore, glycosites often occur in regions of proteins that can be undersampled with standard proteomic methodologies that rely on trypsin. Multiple protease approaches have already proven their benefit for glycoproteome characterization (86, 460, 461, 462, 463, 464). Even so, these largely rely on canonical proteases like trypsin, chymotrypsin, GluC, and AspN, among others. Some classes of glycosylation, *e.g.*, mucin-type O-glycosylation remain impervious to these proteases. Nonspecific proteases can provide benefits in many cases (238, 281, 465), but data analysis of glycopeptides from nonspecific proteolysis can prove challenging and sometimes unreliable. Studies describing proteases that specifically target O-glycoproteins are beginning to emerge (301, 302, 466, 467, 468), which have the potential to make O-glycoproteomics more tractable despite a current lag behind N-glycoproteome characterization. Of importance, inactive point mutants of these proteases (similar to engineered glycosidases discussed above) can also be valuable tools for selective enrichment of the O-glycoproteome (304, 469).

Even the best-suited enrichment methods lack utility if the glycopeptides they enrich cannot be readily identified with the available MS acquisition and informatics tools. Furthermore, enrichment methods cannot be honed or optimized without proper analysis pipelines to facilitate rapid and informative evaluation of outcomes. Tandem MS methodology is paramount to glycopeptide identification and glycosite characterization, and many efforts have explored appropriate fragmentation methods for N- and O-glycopeptides. Collision-based fragmentation, *e.g.*, beam-type collisional dissociation (sometimes referred to as higher-energy collisional dissociation, HCD), is ubiquitous in MS-based proteomics and is indispensable in glycoproteomics, especially for N-glycopeptides. Stepped collision energy methods have become popular for N-glycopeptides, as they balance both glycan and peptide backbone fragmentation within the same MS/MS spectrum (470, 471, 472, 473). Collisional dissociation of O-glycopeptides can still produce glycopeptide identifications, but O-glycosite localization is much more difficult relative to their N-glycosite counterparts. Alternative fragmentation methods, namely, electron-driven dissociation techniques like electron transfer dissociation (ETD) (474) and electron capture dissociation (ECD) (475), are generally needed for O-glycosite characterization because peptide backbone fragments from these methods can retain glycan modifications to pinpoint glycosylated residues even in sequences where many potential glycosites exist. Many modern glycoproteomic methods capitalize on real-time combinations of HCD and ETD scans within the same LC-MS/MS analysis, generally conducted through product-dependent triggering where glycan-specific oxonium ions in “scout” HCD scans trigger subsequent ETD scan(s) for the same precursor ion (476, 477, 478, 479). In addition, ETD and ECD methods often benefit from hybrid approaches that use supplemental activation either during or following the electron-driven dissociation event to promote more extensive fragmentation (480, 481). These hybrid methods have been shown to improve both N- and O-glycopeptide characterization (320, 482, 483, 484, 485, 486, 487, 488), but they are effectively required for site-specific analyses of O-glycopeptides (52, 113, 114, 305, 473). As these nuances between N- and O-glycopeptides continue to be investigated, new acquisitions schemes, such as data-independent acquisition methods (489, 490, 491, 492, 493, 494), are beginning to emerge that have the potential to enable consistent and reproducible characterization of larger and larger subsets of the glycoproteome. Negative-mode approaches that enable analysis of glycopeptide anions are also valuable (495) and will likely contribute to improved characterization of sialylated, sulfated, and otherwise acidic glycopeptides as methods continue to improve (496, 497, 498, 499).

Informatics tools to interpret information-rich glycopeptide fragmentation spectra are equally as essential. Glycosite microheterogeneity makes glycopeptide spectral analysis a far more intricate challenge than other PTMs. Search algorithms must be able to consider a larger number of potential glycoforms for each peptide based on glycan composition libraries that function as variable modifications. This search space issue is even more pronounced for O-glycopeptides, which often harbor multiple glycosylated residues among several potential glycosite candidates. A growing list of academic and commercial software is making glycoproteomics more accessible than ever (65, 66, 67, 68, 69). Among the most popular are Byonic (commercial) and pGlyco (academic), each with their own strengths (224, 500). Particularly exciting are the recently described open-search approaches that not only are more flexible with respect to the glycan composition databases required for analysis (208, 501, 502, 503) but also have the ability to greatly improve spectral processing speeds, a major concern when managing many possible glycoforms considerations. Informatic workflows will also benefit from the incorporation of retention time metrics, which has recently been shown to improve N-glycopeptide assignment (504).

As data acquisition and spectral identification methods mature, quantitative strategies that have benefited MS-based proteomics for nearly 2 decades are beginning to make inroads to enable quantitative glycoproteomics (505, 506, 507). Label-free quantitation is the most widely used quantitative strategy in standard proteomics and has shown utility for glycoproteomics. That said, reproducibility of sample preparation and enrichments are paramount for label-free quantitation approaches, a challenge that is still being addressed in glycoproteomic workflows (508). As such, stable isotope labeling strategies, including metabolic labeling and isobaric labeling, may prove particularly useful as quantitative glycoproteomics comes of age (63). Studies to investigate best practices for isobaric label-centric quantitative glycoproteomic data are emerging, including how to effectively choose ions for synchronous precursor selection for minimal coisolation interference in multiplexed experiments (509, 510, 511, 512). The quantitative approaches selected for glycoproteomics experiments will continue to forge both data acquisition strategies to best acquire accurate quantitative data and also the informatic pipelines to extract and compare quantitative information.

## Glycoproteomics Without Enrichment

Indeed, it is possible to characterize glycopeptides without enriching them first, which would eliminate some of the compromises required when choosing enrichment methods. Glycopeptides are detectable in standard proteomics runs where no enrichment has been performed, even in complex lysates. One simple test to evaluate the presence of glycopeptides in a sample is to generate an extracted ion chromatogram for 204.0867 m/z, the characteristic HexNAc oxonium ion. The product-dependent triggering methods described above (476, 477, 478, 479) can be particularly valuable in these cases to facilitate collection of MS/MS scans with longer ion accumulation times, specific dissociation parameters, and higher-resolution settings, as needed, upon the observation of pertinent oxonium ions. That said, the majority of studies that forgo enrichment characterize relatively simple mixtures of glycopeptides, *e.g.*, glycopeptide standards or glycopeptides derived from purified standard glycoproteins or some form of protein-level enrichment prior to proteolysis (for an example among several, see reference 475). An optimistic outlook suggests that gas-phase enrichment strategies described above, including data-independent acquisition methods, may mitigate some need for preacquisition enrichment in more complex studies, but more work is required to realize such a goal.

## Conclusions

There is currently not a single enrichment method that fully captures the diversity of the glycoproteome. This contrasts with PTMs like phosphorylation or acetylation, where enrichments suffer less from inherent biases. The collection of enrichment strategies described here captures the breadth of approaches needed to interrogate the many facets of protein glycosylation, and even this work is not exhaustive. The size of the analytical toolkit for glycoproteome characterization reflects its heterogeneity and intricacy, which both challenge what biological insights can currently be gleaned in glycoproteomic experiments and also drive innovation. Rather than attempt to capture all that exists in the purview of glycosylation in a single experiment, researchers must continue to judiciously pursue questions about glycobiology that can be honed with specific strategies. As described throughout, combinations of enrichment methods can be creatively synthesized when understanding the strengths and limitations of each approach. The coming years are poised to offer rapid development of glycoproteomic technology. We expect that developments in glycopeptide and glycoprotein enrichment strategies will match the advances of ever-improving instrumentation and software and that exciting new enrichment modalities will continue to drive insight into glycoproteome regulation.

## Conflict of interest

The authors declare that they have no conflicts of interest with the contents of this article.
